# Geographic Realities of Abortion Access in Texas: Exploring the Heterogeneous Effects of Texas Senate Bill 8 with Mobile Phone Data

**DOI:** 10.1007/s11113-025-09948-0

**Published:** 2025-05-05

**Authors:** Jessica Miller, Guangqing Chi

**Affiliations:** Department of Agriculture Economics, Sociology, and Education, Population Research Institute, and Social Science Research Institute, Pennsylvania State University, University Park, PA USA

**Keywords:** Abortion Access, Maternal health, Abortion policy

## Abstract

Restrictive abortion policies have generated reductions in abortion access, increased travel distance to abortion clinics as a result of clinic closures, and produced declines in maternal health outcomes. This study explores the effects of Texas Senate Bill 8 (SB8), the most restrictive bill prior to the overturning of *Roe v. Wade*, on abortion access in Texas. We used a difference-in-differences approach to explore the heterogeneous effects of SB8 on abortion access for communities of varying socioeconomic statuses and travel distances using 16 months of SafeGraph Inc. mobile phone pattern data for 21 Texas and four Oklahoma abortion clinics between January 1, 2021, and April 30, 2022. Implementation of SB8 was associated with 34% fewer abortion clinic visits in Texas than in Oklahoma. The effects of SB8 on access to abortion care across state borders had a disproportionately greater impact on women in low-income communities. This study provides further evidence of the discriminatory impacts of SB8 in Texas.

**Supplementary Information:**

The online version contains supplementary material available at 10.1007/s11113-025-09948-0.

## Introduction

On June 24, 2022, the United States Supreme Court overturned *Roe v. Wade* and sent the determination of abortion rights back to the states in its *Dobbs v. Jackson Women’s Health Organization* decision. Following that decision, 14 states banned or severely restricted abortion care as of March 2023, which increased the number of women of reproductive age living at least 200 miles away from the nearest abortion clinic from approximately 716,000 to 17,000,000, an increase of 2,274% (Bui et al., 2022; Myers, 2021, 2024). Restrictive abortion laws and policies already result in difficult and unequal access to abortion in the United States (Austin & Harper, 2018; Bearak et al., 2017; Lindo & Pineda-Torres, 2021; Myers, 2021). These restrictive laws hinder women from accessing maternal health services as a result of clinic closures, producing increases in travel distances for women seeking such services (Barr-Walker et al., 2019; Bearak et al., 2017; Myers, 2021).

Prior research has focused on the differential fertility outcomes that occur from the burden placed on women by supply-side abortion restrictions (Austin & Harper, 2018; Myers, 2021). Supply-side abortion policies are those that regulate abortion providers through licensing requirements and place gestational caps on abortion access. On September 1, 2021, before *Roe v. Wade* was overturned, the State of Texas implemented Senate Bill 8 (SB8), which prohibits abortion at any time after the detection of fetal cardiac activity and allows any person to take civil or private action against anyone who has an abortion after that time or who helps someone else access abortion care (National & Pillars 2021). SB8 has resulted in a 3% rise in live births and a continued rise in maternal mortality attributed to the reduction in abortion access (White et al., 2022). However, the magnitude of the differential effects of SB8 on abortion access across communities of varying socioeconomic statuses and travel distances to abortion providers has not been measured with rigorous scientific approaches.

The increase in travel distance to clinics associated with the implementation of restrictive abortion laws has historically created declines in abortion rates (Bearak et al., 2017). In Texas, a decline in access caused by increased travel distance over the past decade, linked to an increase in Targeted Regulations of Abortion Providers (TRAP) policies, was estimated to prevent 20.5% of women from seeking an abortion since 2009 and led to a 12.7% reduction in abortions (Myers, 2021). TRAP policies target abortion clinics and providers through admitting-privilege requirements, hospital transfer policies, and facility requirements (Austin & Harper, 2018). A 2013 Texas supply-side policy, House Bill 2 (HB2), which targets providers, led to greatly diminished access as a result of clinic closures. Consequently, travel distances increased, particularly for those living in rural areas (Barr-Walker et al., 2019; Baum et al., 2016; Bearak et al., 2017; Gerdts et al., 2016; Kelly, 2016). One study found that increased travel distance to abortion providers produced nonlinear declines in abortions (Cunningham et al., 2017). The decline in the number of clinics between 2012 and 2014 associated with the passing of HB2 led to an increase from 10 to 44% in the number of women who had to travel over 50 miles to access such care (Dehlendorf et al., 2013; Gerdts et al., 2016; Thompson et al., 2021). Overall, evidence to date points to a relationship between the enactment of restrictive abortion policies in Texas and a deepening of unequal access to abortion care, exacerbated by increased travel distances for substantial numbers of women. The declines in abortions likely indicate that many women were forced to carry a pregnancy they had wanted, or needed, to abort.

Variations in abortion access and travel distances exacerbate existing inequalities in healthcare access. Regardless of distance, abortion restriction, stigma, and financial constraints also make it difficult for women to access abortion services (Bearak et al., 2017). Women of lower socioeconomic status, women living in rural areas, those with second trimester pregnancies, and younger women are more likely to face longer travel distances to clinics, and thus they face additional hurdles in obtaining care (Barr-Walker et al., 2019). Long travel distances and restrictions such as waiting periods often require abortion-seeking women to arrange transportation and childcare, work overtime to afford the cost, and take leave from work, as well as face the social stigma of receiving an abortion (Baum et al., 2016; Kelly, 2016). While access to abortion services is diminished by high travel distances for all women, research shows the effect on abortion care and maternal healthcare is particularly pronounced for young and non-Hispanic Black women (Dehlendorf et al., 2013; Treder et al., 2023).

Initial findings on the impacts of SB8 show reduced abortion rates in Texas, increased waiting time in neighboring states, and increased requests for medical abortions (Aiken et al., 2022; Redd et al., 2021). In the first month after the implementation of SB8, the number of surgical abortions in Texas declined by 50% (Redd et al., 2021). One study found a 10% reduction in travel to abortion clinics in Texas associated with SB8 (Andersen et al., 2023). However, the effects of SB8 on mobility to abortion clinics across socioeconomic status and travel distance remain unknown. This research used SafeGraph mobile phone pattern data to explore the effects of SB8 on abortion clinic access, in terms of the change in clinic visits in Texas. SafeGraph pattern data is a deidentified monthly panel of cellphone data containing information on visitors’ census block group origins to a specific point of interest location. SafeGraph mobile phone data has been used to study the spread of COVID-19 but has been underutilized in other areas of public health research. Specifically, we explored the potential effects at the census block group associated with the implementation of Texas SB8 on visits to facilities that perform abortion and how a potential change in visits varies across levels socioeconomic status and travel distance.

The implementation of SB8 decreased the number of women eligible to receive an abortion through a licensed medical provider in the state of Texas. According to research, the median gestational age at detection of a pregnancy is 5.5 to 6 weeks, which is about the same time frame as the average detection of fetal cardiac activity (Baum et al., 2016). Owing to the limitations placed on abortion providers in Texas through SB8, women might choose or be forced to travel across state borders to seek an abortion. Others may be limited by time or financial constraints and not be able to make a trip across the state border. This scenario would reduce visits to abortion clinics as a result of women in that gestational time frame not seeking treatment through a traditional provider and/or women traveling to an out-of-state abortion clinic with a higher gestational age limit. Overall, we hypothesized that SB8 will significantly reduce clinic access in Texas. We further hypothesized that the reduction in clinic visits as an effect of SB8 falls disproportionality on communities with lower socioeconomic status and those with longer travel distances to abortion clinics.

This study adds to the growing literature on abortion access and policy using innovative big data methods and examines Texas’s new legal approach to anti-abortion laws through civil-oriented policy. With numerous states enacting similar restrictive laws, it is important to explore the impacts on access to abortion care.

## Methods

### Data

To explore the effects of SB8 in Texas, we utilized three datasets: the Advancing New Standards in Reproductive Health (ANSIRH) abortion facility database from the University of California, San Francisco; SafeGraph mobile phone pattern data; and 2015–2019 American Community Survey (ACS) estimates. Below, we provide details about each dataset and their relevance to the study.

### Advancing New Standards in Reproductive Health (ANSIRH) Database

The ANSIRH abortion facility database provides comprehensive and up-to-date information on the location of abortion facilities across the United States. For this study, we used their 2020 database, which includes data on more than 700 abortion clinics nationwide. Of these, 21 were located in Texas and four in Oklahoma. Clinics that were closed or no longer providing abortion services as of January 1, 2021, were excluded from the analysis.

### SafeGraph Mobile Phone Pattern Data

SafeGraph mobile phone data is derived from anonymized location-sharing applications on mobile devices. SafeGraph collects this data through partnerships with mobile app developers and communication companies, creating a large panel of movement data. The dataset includes information about points of interest (POIs)—specific physical locations where consumer or social activities occur—such as retail stores, restaurants, hospitals, parks, and abortion clinics. For this study, POIs represented abortion facilities. SafeGraph provides aggregated data for each POI, which includes monthly visitor counts (phones) to each location and the origin census block groups of visitors (phones).

The dataset covers a broad sample of mobile devices in the United States, estimated to represent approximately 10% of the population (SafeGraph, 2023). SafeGraph provides a monthly panel of mobile phones. Although the dataset does not capture all mobile devices, it offers robust insights into mobility trends. SafeGraph employs strict privacy measures, including noise injection, randomization, thresholds, and differential privacy techniques, to ensure user anonymity (SafeGraph, 2023). However, potential biases may arise, such as overrepresentation of urban or younger populations. Researchers address these biases by comparing the geographic and demographic characteristics of the SafeGraph panel to population benchmarks (e.g., census data) and weighting data accordingly (Hou et al., 2021; Li et al., 2024). For this study, we weighted visitor counts by the monthly panel size and the proportion of women aged 15–44 in each census block group to improve population representativeness (Eq. 1).

For our analysis, we matched abortion clinic addresses from the ANSIRH database to SafeGraph’s POI dataset, achieving a 96% match rate nationwide. This process ensured that only facilities providing abortion services were included. The resulting dataset allowed us to analyze aggregated visitor patterns to abortion clinics at a monthly level, providing key insights into how mobility changed following the enactment of SB8.

### American Community Survey (ACS) Data

To contextualize the mobility data, we used five-year estimates from the 2015 to 2019 American Community Survey (U.S. Census Bureau, 2021). The ACS provides detailed demographic, social, and economic data at the census block group level. This data was used to examine variations in clinic visits across socioeconomic groups and control for key variables such as income, race, education, and poverty.

The main dependent variable was monthly visits to abortion clinics at the census block group. We analyzed abortion clinic visits from Texas census block groups to Texas abortion clinics for eight months before and eight months after the implementation of SB8 (January 2021 through August 2021 and September 2021 through April 2022). We created a comparison trend from clinic visits in the neighboring state of Oklahoma, which had a similar legal gestational age limit to Texas for abortion prior to SB8. We weighted clinic visits by the monthly panel and the proportion of women ages 15 to 44 years to create a population representative sample (Eq. 1). Specifically, monthly visitorship to abortion clinics was aggregated at the census block group and weighted by the corresponding panel per women of reproductive ages, 15 to 44, and multiplied by the raw number of abortion clinic visits (Eq. 1). Weighted visits were then merged with socioeconomic and demographic characteristics from the ACS at the census block group to examine variation in clinic visits across socioeconomic status. Cross-border visits to abortion clinics were removed, so the dataset contained visits only to Texas abortion clinics from Texas census blocks group and visits only to Oklahoma clinics from Oklahoma census blocks group. Separately, a dataset was created of visits from Texas census blocks to both Oklahoma clinic and Texas clinics to examine change in cross-border clinic visits of Texans to Oklahoma, across income groups. Although Texans traveled to states other than Oklahoma for abortion care, Oklahoma was specifically used in this study because of its similar legal environment to Texas prior to SB8 implementation (see supplement for parallel trends analysis).

1 $${\text{Monthly visitorship}} = {\text{Raw Clinic Visits}} \times \frac{{\text{Visitor Panel}}}{{{\text{Women of Reproductive Ages}}, 15 - 44}} \times 100$$

**Fig. 1:**
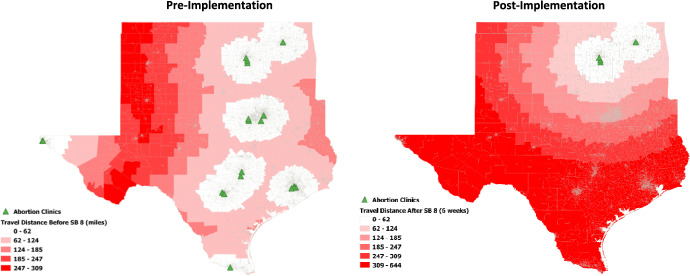
Average distance to abortion clinic pre- and post-SB8

The main independent variables used in this study were travel distance and median income at the census block group. We calculated the average euclidean travel distance from census block centroids to the nearest abortion clinic in the open-source QGIS system before and after implementation of SB8. Travel distance ranged from 0 to 308 miles, with a mean of 46.9 miles. Low, medium, and high travel distance indicators of 30 miles or less, 30 to 70 miles, and 70 miles or more were created to examine compounding barriers to abortion access. Travel distance after implementation of SB8 was calculated from each Texas census block to the nearest Oklahoma abortion clinic. Travel distances after implementation ranged from 0 to 644 miles, with a mean of 275 miles (Fig. 1). Similarly, low-, medium-, and high-income variables were created to examine variation in clinic visitorship. Low-income census blocks were defined as those with a median household income of less than $25,000. Medium income was defined as $25,000 to $65,000. High income was defined as household median income over $65,000. The income categories were created using the interquartile range, where census blocks with incomes 25% or lower than the average were designated low income, 25 to 75% (or the median income) as medium income, and 75% or higher as high income.

Research has shown that poverty, race, and level of education are correlated with abortion access and mobility (Baum et al., 2016; Solazzo, 2019). Therefore, to identify the effect on clinic visits associated with SB8, we controlled for each of those variables (Table 1). We controlled for educational attainment, poverty level, percentage of the female Hispanic population, percentage of the female Black population, and percentage of the female White population at the census block group. Educational attainment was measured as the average percentage of the population aged 25 years and above with at least a bachelor’s degree. Race was measured with three variables: the percentage of the Black population, the percentage of the White population, and the percentage of the Hispanic population at the census block group. The poverty level was measured as the percentage of households in a census block living in poverty. All variables were derived from ACS estimates.

**Table 1:** Descriptive statistics for variables included in the difference-in-differences regression model

	SD	Mean or %	Min	Max
Variables				
Abortion clinic visits	308	14	0	158,042
State (1 = TX, 0 = OK)			0	1
SB8 implemented (1 = yes, 0 = no)			0	1
% White female	9	7	0	100
% Black female	5	2	0	83
% Hispanic female	5	4	0	90
% Female with bachelor’s degree	14	18	0	100
Female income	$16,119	$26,367	$0	$250,000
% households in poverty	13	16	0	100
Travel distance before SB8	63	48	0	309
Travel distance after SB8	159	275	0	644
Month	5	8	0	15

Variables included in the difference-in-differences fixed-effect regression models

## Methodology

First, we examined abortion clinic visits across varying levels of income and travel distances in Texas and Oklahoma pre- and post-implementation of SB8, as well as the percentage change in in-state and out-of-state clinic visits for people traveling from Texas census blocks. Then we utilized a difference-in-differences model to assess the initial effects of SB8 on clinic access in Texas relative to Oklahoma. Mobility to abortion providers in Oklahoma was used as a comparator because of the legal environment that existed before the passing of SB8—Oklahoma and Texas had similar TRAP laws that prohibited abortion beginning at around 20 weeks of pregnancy (Barr-Walker et al., 2019). Visitors from census blocks in Oklahoma, where SB8 was not in effect, served as the control group. Clinic visits pre- and post-implementation and visits in Oklahoma and Texas were both dummy variables. The indicator for the presence of SB8 and time of implementation were multiplied to estimate the effect of SB8 on clinic visits in Texas. After running the full model, we tested income and used distance stratified models to examine the variation in magnitude of the impact of SB8 on visitation to abortion clinics. The income and distance models were stratified by the categorical income and distance variables, respectively, described in the data section of this paper.

2 $$y\left( {{\text{ClinicVisits}}} \right) = b + b1\left( {{\text{Time}}} \right) + b2\left( {{\text{Treatment}}} \right) + b3\left( {\text{DiD Estimator}} \right) + b4\left( {\text{Vector of Controls}} \right) + e$$

whereTime: 0 = pre-implementation of SB8, 1 = post-implementation of SB8.Treatment: 0 = Oklahoma, 1 = Texas.The difference-in-differences estimator is a dummy created by multiplying the timing of implementation by the treatment, whether or not SB8 was present, where 1 means SB8 was implemented in Texas and 0 means SB8 was not implemented—i.e., in the control state (Oklahoma).Vector of controls include the percentages of the White female population, Black female population, Hispanic female population, female population 25 and older with a bachelor’s degree, and households in poverty, as well as travel distances and female income before implementation and after implementation.Fixed variables include panel month, where January 2021 = 0 and April 2022 = 15.*e* represents residuals.

### Role of Funding Source

The funders of the study had no role in study design, data collection, data analysis, data interpretation, or writing of the manuscript.

## Results

### Change in In-State and Out-of-State Clinic Visits Among Texas Communities

Consistent with previous research, we found that women from low- and medium-income communities made the most abortion clinic visits (10% and 19%, respectively). They experienced a respective 30% and 40% increase in visits to Texas clinics after SB8 was implemented—likely owing to the lifting of healthcare pandemic restrictions (Table 2). On the other hand, high-income areas had only a 3% increase in in-state clinic visits. In that same period, there was a reduction of 4% in the number of Texans from low- and medium-income communities visiting Oklahoma clinics. Their counterparts from high-income areas, however, showed a 144% increase in out-of-state abortion clinic visits. It is likely that people with greater financial means, better access to transportation, and wider social networks had a greater ability to travel across state lines to obtain abortion care.

**Table 2:** Change in Texas female abortion clinic visitation to clinics in Texas and Oklahoma after the implementation of SB8

	Texas Clinics	Oklahoma Clinics
Income group		
Low	30%	–4%
Median	40%	–4%
High	3%	144%

Percentage change in abortion clinic visits between Jan 1, 2021–Aug 30, 2021, and Sept 1, 2021–Apr 30, 2022

### In-State Clinic Visits and Travel Distance

The number of abortion clinic visits varied across travel distance. After the implementation of SB8, areas in Texas with travel distances of less than 30 miles to abortion clinics had a 4.3% rise in clinic visits, whereas areas with a high travel distance (more than 70 miles) had a 31.7% reduction in clinic visits. The average woman from a community farther away from an abortion clinic experienced an 830.4% greater reduction in abortion access than an average woman from communities within a 30-mile radius of an abortion clinic. Areas with high travel distances and low income in Texas were further disadvantaged. There was a 26.5% decline in abortion clinic visits for women traveling from low-income areas more than 70 miles away from a clinic. High-income areas with low travel distances had a 47.9% decline in abortion clinic visits. The results reflect that an uneven reduction in clinic-based abortion access and immediate closures in wealthier urban centers could possibly be attributed to disparities across place and economic status.

### Associated Effects of Texas Senate Bill 8

The results of a difference-in-differences model showed a significant reduction in abortion clinic visits associated with SB8 in Texas (Eq. 2). With everything else held constant, the implementation of SB8 was associated with a significant percentage (33.7%) of fewer abortion clinic visits in Texas relative to Oklahoma. There was also a significant percentage (62.8%) of fewer clinic visits associated with Texas compared with Oklahoma, with everything else held constant (Table 3). Overall, the post-implementation period was associated with a 27.0% rise in clinic visits, likely caused by the ending of pandemic restrictions on abortion care (Table 3).

**Table 3:** Average abortion clinic visitation before and after SB8, by income

Overall Effect SB8 on the Population			
	TX	OK	Difference
Before	1.84%	62.52%	–60.68%
After	1.86%	92.52%	–90.66%
Difference	0.02%	30.00%	–29.98%

Effect of SB8 on Low-Income Population			
	TX	OK	Difference
Before	2.14%	45.08%	–42.94%
After	4.47%	60.87%	–56.40%
Difference	2.33%	15.79%	–13.46%

Effect of SB8 on Median-Income Population			
	TX	OK	Difference
Before	2.88%	85.81%	–82.93%
After	4.68%	124.03%	–119.35%
Difference	1.80%	38.22%	–36.42%

Effect of SB8 on High-Income Population			
	TX	OK	Difference
Before	37.49%	86.06%	–48.57%
After	0.00%	172.71%	–172.71%
Difference	–37.49%	86.65%	–124.14%

Abortion clinic visitorship was associated with varying socioeconomic outcomes (Table 4). When holding everything else constant, income had a statistically significant positive association with abortion clinic visits. In other words, women from high-income areas were, on average, associated with higher abortion clinic visits compared with women from low-income areas. When exploring how income moderates the effect of clinic visits related to SB8, the opposite relationship appears. The predicted effects show that, on average, women from high-income areas made fewer abortion clinic visits compared with women from low-income areas, when everything else is held constant (Table 5). The implementation of SB8 was associated with an 87.7% reduction in abortion clinic visits among high-income women in Texas, a 38.2% reduction for medium-income women, and a 15.75% reduction for lower-income women when controlling for other factors (Table 5).

**Table 4:** Associated effect of SB8 on abortion clinic visitation in Texas January 2021–August 2021 and September 2021–April 2022

	Model 1		Model 2		Model 3	
Main variables						
State (1 = TX, 0 = OK)	–63.66	0.00	–63.25	0.00	–63.30	0.00
SB8 implemented (1 = yes, 0 = no)	44.32	0.00	44.31	0.00	44.31	0.00
State: SB 8 implemented (DiD estimator)	–27.00	0.00	–27.00	0.00	–33.72	0.00
Control variables						
% White female			0.22	0.00	0.22	0.00
% Black female			–0.19		–0.19	
% Hispanic female			–0.34	0.00	–0.35	0.00
% Females with bachelor’s degree			–0.03		–0.03	
Female income			0.00	0.00	0.00	0.00
% Households in poverty			–0.03	0.00	–0.35	0.00
Travel distance before SB8			–0.05	0.00	–0.07	0.00
Travel distance after SB8			0.00		0.00	
Interaction variables						
DiD estimator: travel distance before SB8					0.06	0.00
DiD estimator: female income					–0.00	0.01
DiD estimator: % households in poverty					0.56	0.00
Fixed effect	Yes		Yes		Yes	
N	300,282		300,282		300,282	
Adjusted R-squared	0.01	0.00	0.01	0.00	0.03	0.00

*DiD stands for difference-in-differences

**Table 5:** Associated Effect of Texas Senate Bill 8 on abortion clinic visitation in Texas between January 2021–August 2021 and September 2021–April 2022 by Income

	Low income		Middle income		High income	
Main variables						
State (1 = TX, 0 = OK)	−42.89	0.00	−80.70	0.00	−90.06	0.00
SB8 implemented (1 = yes, 0 = no)	27.37	0.00	61.45	0.00	96.74	0.00
State: SB8 implemented (DiD estimator)	−15.75	0.00	−38.16	0.00	−87.71	0.00
Control variables						
% White female	0.07	0.01	0.95	0.00	−0.26	0.00
% Black female	−0.09		−0.37		−0.14	
% Hispanic female	−0.02		−1.20	0.00	0.39	0.01
% Females with bachelor’s degree	−0.03		−0.00		−0.24	0.00
% Households in poverty	−0.18	0.00	−0.57	0.00	−0.55	0.00
Travel distance before SB8	−0.03	0.00	−0.06	0.00	0.01	
Travel distance after SB8	−0.00		0.00		0.01	
Fixed effect	Yes		Yes		Yes	
N	147,071		147,846		4,800	
Adjusted r-squared	0.03	0.00	0.01	0.00	0.10	0.00

Consistent with previous research, we found that travel distance from abortion clinics was associated with a decline in abortion clinic visits. Every 10-mile increase in distance to the nearest abortion clinic was associated with a 1% reduction in abortion clinic visits, with everything else being held constant: women from census blocks the farthest away from abortion clinics before the implementation of SB8 made fewer clinic visits than women residing in census blocks closer to abortion clinics. As with income, the effects of SB8 on clinic visits resulted in varying outcomes across communities with different travel distances to abortion clinics. The effect of SB8 on Texas women from census blocks farther away from abortion clinics post-implementation was associated, on average, with higher clinic visits compared with those women who had lower travel distances to abortion clinics that observed lower clinic visitorship. Unlike communities with long travel distances to clinics before SB8, the average increase in distance to an abortion clinic after implementation showed a nonsignificant association (0.02%) with abortion clinic visits, with everything else being held constant. We did not find a significant difference in the associated effect of SB8 on visitation across census blocks with low (30 miles away), medium (30 to 70 miles away), and high travel distance (more than 70 miles away). This is likely due to a reduction in abortion care, mainly in urban centers.

## Discussion

In the period after the passing of Texas SB8, visits to abortion clinics in the state of Texas declined significantly. This corresponds to a reported decline of 50% in the abortion rate in that state in September 2021 (Redd et al., 2021). The immediate effects of SB8 were felt by women of all communities. It particularly impacted high-income, urban women who experienced local clinic closures; and low-income, rural women without the financial means and networks to travel across state borders to obtain a surgical abortion. Though the largest reductions in abortion access occurred among women from high-income urban areas, cross-border travel to abortion clinics increased in those areas, whereas travel to in-state abortion clinics from women living in farther-away rural areas increased. SB8 likely compounded the effects of previous clinic closures in rural and remote areas from bills such as HB2.

Access to abortion clinics contrasted strongly between high-income and low-income areas. There were disproportionate increases for Texas women with a median household income of over $65,000 who traveled to Oklahoma after the passing of SB8. We found a 59% increase in Texas residents traveling to Oklahoma abortion clinics immediately following the implementation of SB8. Abortion travel for low- and medium-income Texas residents declined by around 4%, compared with a 144% increase among high-income Texans. The changes in abortion access for Texas residents disproportionately jeopardize the lives of women who cannot afford to travel for medical care, and the overturning of *Roe v. Wade* will likely have a much larger impact on outcomes for low-income women.

### Health Implications

The evidence from Texas shows that abortion restrictions and gestational limits do not prevent everyone from having an abortion. Individuals with resources to circumvent the hurdles will still be able to get the abortions they need. Wealthier women travel to obtain abortions, and those who cannot afford to travel search for other options (Aiken et al., 2022). Research has found that communities of color, particularly Hispanic and Black communities, have a higher rate of maternal mortality (Addante et al., 2021; Nambiar et al., 2023). Pregnancy is a greater risk to women’s health than abortion, and these legal changes will cause more women (particularly non-Hispanic Black women) to be forced to carry a pregnancy; such women are also two to three times likelier to die from pregnancy-related complications compared with non-Hispanic White women (Mosley et al., 2022). Data show that states with more-restrictive abortion policies experience higher maternal mortality rates (Addante et al., 2021).

The overturning of *Roe v. Wade* has already decreased abortion access for millions of people in the United States. States with a restrictive abortion environment similar to that in Texas, or a complete ban, are likely to see more hospitalizations from unsafe abortions and risky pregnancies, as well as increasing levels of poverty and inequality in their states. Abortion bans will increase travel dist.

ance to abortion clinics, and, as already seen in Texas, disproportionately harm low-income communities of people who might lack the means to travel for healthcare (Miller et al., 2022). Women who have the resources to travel for an abortion are able to exercise their right to reproductive autonomy, to have a child if and when they desire, and to protect their health and well-being. Women without those resources bear the brunt of restrictive abortion policies that obstruct their right to the same choices. We recommend that future research address the long-term effects of restrictive abortion policies on health and well-being, as well as how abortion access is related to economic inequality.

### Limitations

This research used panel cellphone data to study abortion clinic visits; however, we do not know whether the women obtained abortions or were turned away, nor do we know whether a companion went with them to the clinic. All of the abortion facilities in our dataset provided abortion procedures. However, many of them also provide a wide range of other women’s health services, from pap smears to birth control. It is not known what services the women who visited these clinics sought or received.

## Supplementary Information

Below is the link to the electronic supplementary material.

Supplementary file1 (DOCX 136 KB)

## Data Availability

The ACS data used in this study are publicly available. For the ANSIRH abortion facility database and the mobile phone data, a request should be made to the University of California San Francisco and SafeGraph, respectively.
