# Supplementary material for: Geographic Realities of Abortion Access in Texas: Exploring the Heterogeneous Effects of Texas Senate Bill 8 with Mobile Phone Data
Source: Popul Res Policy Rev. 2025 May 5;44(3):29. doi: 10.1007/s11113-025-09948-0 (PMC12053199; doi:10.1007/s11113-025-09948-0)
Supplement: Supplementary file 1 — Supplementary file1 (DOCX 136 KB) [file 11113_2025_9948_MOESM1_ESM.docx]

**Figure 1 Difference in Difference Analysis of Abortion Clinic Visits**


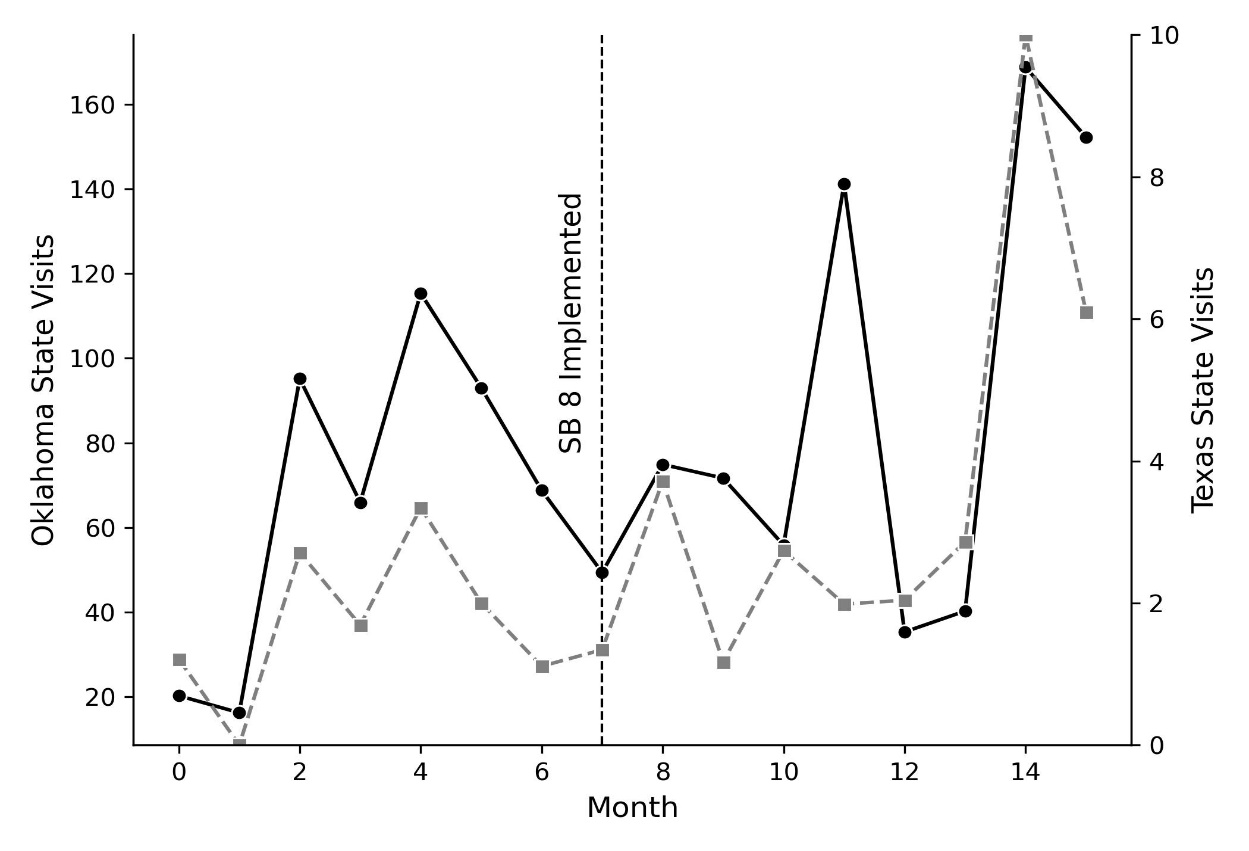


TX Visits

OK Visits

Source: Safegraph Inc. mobile phone data.

Note: The left y-axis of the graph corresponds to Oklahoma (OK) visits and the right y-axis corresponds to Texas (TX) visits. Texas and Oklahoma visits correspond to average aggregated visits to abortion clinics measured by mobile phone data. Due to the numerical difference in average visitation per month, the trend lines were overlayed corresponding to different y-axis. SB 8 stands for Texas Senate Bill 8.

| **Table A. Difference in marginal effects of time on abortion clinic visits in Texas and Oklahoma** | | |
| --- | --- | --- |
|  | Estimate | Std. Error |
| Main Variables |  |  |
| Month (Jan 21-Aug 21) | 6.08*** | 0.48 |
| State (1= TX, 0=OK) | -41.86*** | 3.32 |
| Month: Region | -6.02*** | 0.79 |
| N=37,390 |  |  |
| R-squared= 0.03*** | |  |

Source: Safegraph Inc. mobile phone data.

We tested the parallel trends assumption and found mixed evidence. Specifically, we calculated the monthly aggregated clinic visits in Texas and Oklahoma during the pre-implementation period (figure 1). While visual inspection indicated that the two states exhibited broadly similar trends in monthly abortion clinic visitation before the implementation of SB8, our statistical tests revealed a significant difference in the marginal effect of time on visitation between Texas and Oklahoma (table A). This suggests that the two states did not perfectly trend together in the pre-implementation period, indicating a potential violation of the parallel trends assumption.

In figure 1 and table A we test the assumption of parallel trends. This tests whether Texas and Oklahoma observed similar patterns in abortion clinic visitation prior to the implementation of SB8. Figure 1 presents a visual representation of abortion clinic visitation for the two states during the pre-implementation and post-implementation period. It shows a similar trend between TX and OK before SB8 implementation. After SB8 went into effect, visitation to abortion clinics display a divergent trend.

However, table A in the document presents the difference in marginal effects of time on abortion clinic visitation between Texas and Oklahoma. There was a negative and statistically significant estimate (-6.02, p < 0.001) between the Month × State Interaction (Divergence in Trends), indicating that the time trend in Texas differed from that in Oklahoma in the pre-treatment period. This is a potential violation of the parallel trends assumption.

If the parallel trends assumption was violated, it implies that external factors may have influenced visitation to abortion clinics differently in each state during the pre-treatment period. One key factor likely driving these differences is the COVID-19 pandemic, which had a significant and uneven impact on in-person healthcare across states during this time (Baum et al., 2020; Burke et al., 2022). The pandemic introduced variability in healthcare access and patient behavior, complicating the establishment of a clear and consistent baseline month for analysis.

Despite these challenges, we believe the results presented in this study remain valuable. The observed divergence in visitation trends following the implementation of SB8 aligns with theoretical expectations and provides important insights into how abortion restrictions affect clinic access and mobility patterns. Additionally, this analysis demonstrates the utility of novel data sources for examining complex and dynamic policy impacts. Future research may benefit from further refining approaches to account for external factors such as the COVID-19 pandemic when analyzing the effects of restrictive healthcare policies.
